# Necrotizing fasciitis due to *Vibrio cholerae* non-O1/non-O139 after exposure to Austrian bathing sites

**DOI:** 10.1007/s00508-015-0944-y

**Published:** 2016-01-29

**Authors:** Sonja Hirk, Steliana Huhulescu, Franz Allerberger, Sarah Lepuschitz, Sonja Rehak, Sandra Weil, Elisabeth Gschwandtner, Michael Hermann, Stephanie Neuhold, Alexander Zoufaly, Alexander Indra

**Affiliations:** 1 Institute of Medical Microbiology and Hygiene, Österreichische Agentur für Gesundheit und Ernährungssicherheit (AGES), Spargelfeldstraße 191, 1220 Vienna, Austria; 2 2. Chirurgische Abteilung, Wiener Krankenanstaltenverbund, Krankenanstalt Rudolfstiftung, Vienna, Austria; 3 4. Medizinische Abteilung mit Infektiologie und Tropenmedizin, Wiener Krankenanstaltenverbund, Kaiser Franz Josef Spital, Vienna, Austria; 4 Division for Medical Microbiology, Institute for Laboratory Medicine, Paracelsus Medical University, Salzburg, Austria

**Keywords:** *Vibrio cholerae*, Necrotizing fasciitis, Bathing sites, Climate change, Heat wave, Drought

## Abstract

We report on two cases of necrotizing fasciitis of the lower leg due to nontoxigenic *Vibrio cholerae* (*V. cholerae*). A 73-year-old woman (case 1) and an 80-year-old man (case 2) were hospitalized with symptoms of necrotizing fasciitis on July 18 and August 15, 2015, respectively. In both cases, symptoms started the day after swimming in local ponds. Swabs gained intraoperatively and a blood culture from the male patient, yielded *V. cholerae* non-O1/non-O139, negative for cholera toxin gene *ctx* and positive for hemolysin genes *hly*A and *hly*B. Water samples taken from pond A on August 17, 2015 (32 days after exposure of case 1) and from pond B on August 20, 2015 (7 days after exposure of case 2) yielded non-O1/non-O139 *V. cholerae* in most-probable numbers of > 11,000 per 100 ml each. The occurrence of two cases of necrotizing fasciitis within a 1 month period related to two Austrian non-saline bathing waters, previously not known to harbor *V. cholerae*, is probably linked to the prevailing extreme weather conditions (heat wave, drought) this summer in Austria. While case 1 was discharged in good clinical condition after 73 days, case 2 died after four months of hospitalization. Public health authorities are challenged to assess the effects of long-term climate change on pathogen growth and survival in continental bodies of fresh water.

## Background

*Vibrio (V.) cholerae* is a gram-negative rod-shaped bacterium, which preferentially grows in warm (> 15 °C) brackish and estuarine water [1, 2]. Only serogroups O1 and O139 are known to cause classic cholera [3–5]. The other approximately 200 serogroups of non-O1/non O-139 *V. cholerae* rarely harbor cholera toxin (*ctx*), often carry hemolysin genes *hly*A and *hly*B and usually only cause self-limited gastroenteritis or mild extra intestinal symptoms [3, 6]. We report two cases of necrotizing fasciitis caused by non-O1/non-O139 *V. cholerae*, acquired by swimming at Austrian bathing sites, at the peak of a 2 month long heat period characterized by an extraordinarily low amount of rainfall, in 2015.

## Case reports

### Case 1

**Fig. 1:**
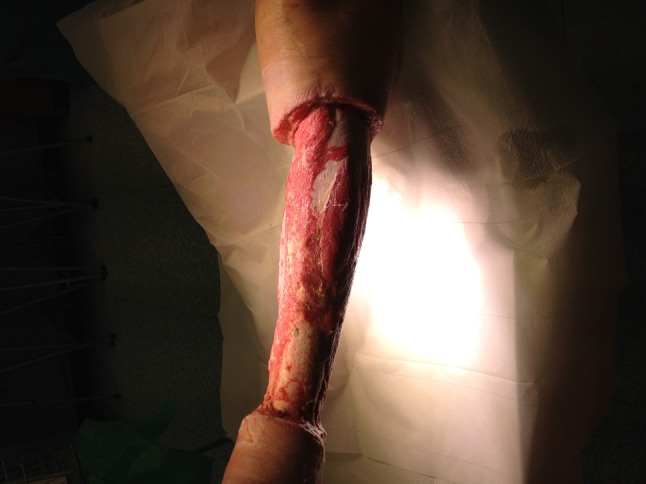
Left lower leg of case 1 on day 14 of hospitalization

**Fig. 2:**
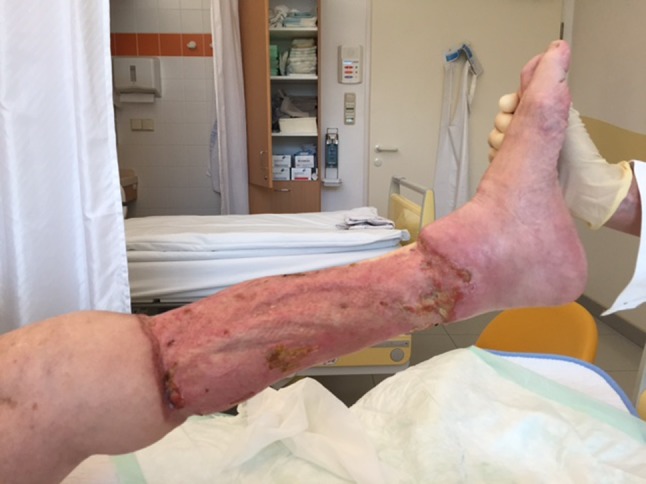
Left lower leg of case 1 on day 68 of hospitalization

On July 18, 2015, a 73-year-old obese woman (body mass index 30.5) with hypertonia and diabetes mellitus presented herself to the emergency room of hospital A in Vienna, one day after swimming in pond A in the province of Lower Austria. She complained of severe pain in the left lower leg, livid-blue discoloration, local hyperthermia and of fever up to 38 °C. She recalled a minor excoriation on her left leg. Laboratory examinations demonstrated elevated C-reactive protein (26.0 mg/l, normal < 5 mg/l), a white blood cell count of 19.0 G/l (normal 4–9 G/l) and elevated serum-lactate (3.4 mmol/l, normal 0.5–1.6 mmol/l). A duplex sonography of the leg veins indicated a compartment syndrome. Initial surgical treatment consisted of bilateral fasciotomy (medial + lateral). Necrotizing fasciitis was diagnosed and swabs gained intraoperatively yielded non-O1/non-O139 *V. cholerae* (bacteriological results reported on day 7). Blood cultures remained sterile. Empiric antibiotic therapy initiated on day 1 consisted of ampicillin/sulbactam (3 g, tid, IV). On day 2 of hospitalization, C-reactive protein increased to 393.6 mg/l and white blood cell count to 26.4 G/l; serum procalcitonin was 33.48 ng/ml (normal < 0.5 ng/ml), antithrombin III activity was 66 % (normal 83–128 %) and the patient required intensive care. Antibiotic treatment was switched to a combination of piperacillin/tazobactam (4.5 g, tid, IV) and fosfomycin (8 g, tid, IV); the first of four soft tissue debridements was performed that day. On day 3, clindamycin (900 mg, tid, IV) was added; antithrombin III activity was 34 % and C-reactive protein 463.2 mg/l. On day 5, the patient became afebrile. On day 7, with arrival of bacteriological results showing *V. cholerae* susceptible to piperacillin/tazobactam, antibiotic therapy was deescalated to the latter antimicrobial for another 2 weeks. The isolates were also susceptible to ampicillin, trimethoprim-sulfamethoxazole, ciprofloxacin and tigecycline, tested according to the European committee on antimicrobial susceptibility testing (EUCAST) recommendations for enterobacteriaceae [7]. Wound swabs taken on day 3, 4, and 6 again yielded *V. cholerae*. The first culture-negative wound swab was on day 9. A stool sample on day 7 was negative for *V. cholerae*. On day 9, negative pressure wound therapy system (VAC, vacuum-assisted closure; KCI Corp, Vienna, Austria) was applied. On day 14, the patient was transferred to a surgical ward. Figure 1 depicts the wound status at that point in time. On day 22, a split skin-graft transplantation was done. The patient was transferred to the plasticsurgery ward on day 27, where she stayed till discharge on September 30, after 73 days of hospitalization. Figure 2 depicts the clinical status as of day 68.

### Case 2

On August 15, 2015, an 80-year-old man presented himself to the emergency room of hospital B in the province Lower Austria, one day after swimming in local pond B. During the summer, the patient had repeatedly used this pond for swimming. He complained of increasing swelling and pain in his left lower leg, fever (38 °C), dyspnea, and malaise since last night. His medical history was unremarkable except for ichthyosis cutis and several episodes of cellulitis. He recalled a minor trauma after hitting his left ankle on a table the day before swimming. The patient was admitted under the presumptive diagnosis of deep vein thrombosis and pulmonary embolism. His vital signs (blood pressure 78/36 mmHg; heart rate 130/min) deteriorated rapidly, and on day 2, by then the severely septic patient was admitted to intensive care unit. Blood cultures taken on day 2 yielded *V. cholerae* (bacteriological results reported on day 5). On day 3, bilateral fasciotomy and debridement were performed on his left lower leg and necrotizing fasciitis was diagnosed; swabs taken intraoperatively yielded growth of non-O1/non-O139 *V. cholerae*. Antibiotic therapy was initiated with piperacillin/tazobactam (4.5 g, tid, IV), tigecycline (100 mg, bid, IV), and metronidazole (500 mg, tid, IV). On day 5, the patient was transferred to an ICU specialized in infectious diseases (Hospital C, Vienna). Laboratory examinations demonstrated elevated C-reactive protein (265 mg/l, normal < 5 mg/l) and a white blood cell count of 35.0 G/l (normal 4–9 G/l). *V. cholerae* (isolated from wound swabs until day 7) was susceptible to ampicillin, trimethoprim-sulfamethoxazole, ciprofloxacin and tigecycline, tested according to the European committee on antimicrobial susceptibility testing (EUCAST) recommendations for enterobacteriaceae [7]. On day 5, blood cultures were positive for *V. cholerae* and antibiotic treatment was switched to piperacillin/tazobactam (4.5 g, tid, IV) in combination with clindamycin (600 mg, tid, IV) and doxycycline (100 mg, bid, IV). Due to suspected ventilator-associated pneumonia, piperacillin/tazobactam was switched to meropenem (1 g, tid, IV); clindamycin was stopped on day 13. A total of five surgical revisions and application of negative pressure wound therapy system (VAC, vacuum-assisted closure, KCI Corp, Vienna, Austria) followed. After 5 weeks of hospitalization, a split skin-graft transplantation was performed. Case 2 died after four months of hospitalization.

## Environmental investigations and subtyping

A total of seven *V. cholerae* isolates (two human isolates, two from pond A and three from pond B) tested by NGS lacked cholera toxin genes *ctx*A, *ctx*B, toxin-coregulated pilus (TCP), heat-stable enterotoxin gene *sto* and NAG-ST gene *stn*. All seven *V. cholerae* isolates were positive for El Tor like hemolysin genes *hly*A and *hly*B.

A water sample taken from pond A on August 17, 2015 (32 days after exposure of case 1) yielded non-O1/non-O139 *V. cholerae* in most-probable numbers (MPN) of > 11,000 per 100 ml tested by MPN method [8] (enterococci: < 15 MPN/100 ml; *Escherichia coli* (*E. coli*): < 15 MPN/100 ml according to ÖNORM EN ISO 7899-1 and ÖNORM EN ISO 9308-3 respectively [9, 10]). Pond A (water-surface area: 20,000 m^2^, maximum depth: 17 m) is a brick pond, located in a village of approximately 5000 inhabitants south of the city of Vienna. It is not registered as an EU bathing site but widely used for swimming.

A water sample taken from pond B on August 20, 2015 (7 days after exposure of case 2) yielded non-O1/non-O139 *V. cholerae* in numbers of > 11,000 MPN per 100 ml (enterococci: 127 MPN/100 ml; *E. coli*: 161 MPN/100 ml). Pond B (water-surface area: 600 m^2^, maximum depth: 3 m) is a former gravel quarry situated 20 km south of pond A. It is not registered as an EU bathing site, but used for swimming by locals.

Both ponds were resampled on September 1, 2015: pond A showed non-O1/non-O139 *V. cholerae* in numbers of 2,400 MPN/100 ml (enterococci: < 15 MPN/100 ml; *E. coli*: < 15 MPN/100 ml), pond B, 11,000 MPN/100 ml (enterococci: 15 MPN/100 ml; *E. coli*: < 15 MPN/100 ml). Salient features of the two swimming sites are summarized in Table 1.

**Table 1:** Summarized data on bathing sites related to non-O1/non-O139 *Vibrio cholerae* necrotizing fasciitis

Sampling date	Bathing site	*V. cholera* non-O1/non-O139 in MPN/100 ml	Patient	Water temperature in °C	pH	Conductivity in µS/cm
Aug 18, 2015	Pond A	> 11,000	Case 1	28.6	8.9	2,320
Aug 20, 2015	Pond B	> 11,000	Case 2	20.5	8.9	1,242
Sept 01, 2015	Pond A	2,400	Case 1	25.2	9.0	2,260
Sept 01, 2015	Pond B	11,000	Case 2	25.0	8.4	1,220

*MPN* most-probable number

On August 17 and 18, a total of 90 of 175 Austrian bodies of water registered as EU bathing sites were tested for *V. cholerae*. Lake Neusiedl (located in the Austrian province Burgenland), three further bodies of water in Burgenland and three in the province Lower Austria tested positive for non-O1/non-O139 *V. cholerae*. Data on positive water samples are summarized in Table 2. With the exception of Asangteich, which showed 534 MPN/100 ml enterococci and 127 MPN/100 ml *E. coli*, all 90 bodies of water showed less than 400 MPN/100 ml enterococci and less than 1,000 MPN/100 ml *E. coli*.

**Table 2:** Summarized data of EU bathing waters harboring *Vibrio cholerae* non-O1/non-O139 in Austria, August 2015

Body of water	Bathing site	Province		*V. cholerae* non-O1/non-O139 in MPN/100 ml	Water temperature in °C	pH
Lake Neusiedl	Weiden		Burgenland	> 11,000	24.9	8.8
	Neusiedl			360	24.6	8.7
	Breitenbrunn			4,600	24.4	8.8
	Rust			4,600	25.5	8.6
	Podersdorf			11,000	23.8	8.9
	Illmitz			> 11,000	24.9	8.9
	Mörbisch			11,000	25.4	8.8
Lake Andau	Lake Andau		Burgenland	930	25.5	8.4
Lake Apetlon	Lake Apetlon		Burgenland	2,400	25.0	8.6
Zicksee	St. Andrä		Burgenland	2,100	21.3	8.9
Ausee	Blindenmarkt		Lower Austria	150	23.1	8.4
Lake Hohenau	Lake Hohenau		Lower Austria	1,500	24.2	9.0
Lake Seeschlacht	Langenzersdorf		Lower Austria	> 11,000	24.6	8.4

*MPN*  most-probable number

## Discussion

In Austria, occurrence of non-O1/non-O139 *V. cholerae* has so far only been known for Lake Neusiedl, a saline steppe lake in eastern Austria, bordering Hungary [3, 4, 11]. Huhulescu *et al*. [3] previously reported the occurrence of human cases of otitis externa, otitis media, mild diarrhea and one fatal case of septicemia in an immunocompromised patient after swimming in this lake; lake Neusiedl has a saline concentration approximately one-tenth of the Mediterranean Sea. In 2015, two cases of non-O1/non-O139 *V. cholerae* infections were documented in relation to Lake Neusiedl: A 21–year-old woman saw an otolaryngologist for otitis externa on August 17, 2015 and a 28-year-old male patient presented himself to an outpatient-clinic for infection of the urogenital tract (“bloody seminal fluid”) on September 3, 2015 (unpublished data).

To our knowledge, the two cases of necrotizing fasciitis due to non-O1/non-O139 *V. cholerae* described here are the first cases documented in Austria. Necrotizing fasciitis due to non-O1/non-O139 *V. cholerae* has previously been reported in the scientific literature. In Europe, one isolated case of necrotizing fasciitis caused by non-O1/non O-139 *V. cholerae* (and associated with water exposure) was reported Italy (Mediterranean sea) [12]. In contrast to our Austrian cases, all those infections were associated with saline waters, as were singular reports of *V. cholerae* non-O1/non O-139 necrotizing fasciitis from the United States [13] and Taiwan [14, 15].

The occurrence of two cases of necrotizing fasciitis within a 1 month period related to two Austrian non-saline bathing sites, previously not known to harbor *V. cholerae*, is probably related to the prevailing extreme weather conditions (heat wave, drought) in Austria during this summer. Austria-wide, temperatures in July 2015 were 3.1 °C higher than the average (mean) measured from 1981 to 2010. It was the warmest July on record since 1767. The rate of precipitation in July 2015 was 20 % below average (as measured from 1981 to 2010) Austria-wide. In Lower Austria, the deviation in precipitation was 41 % below average [16] and temperatures were + 3.2 °C above average in July 2015. August 2015 deviated from the accepted average taken 1981–2010 by + 2.7 °C, the fourth warmest month since records began in 1767; there was 35 % less rainfall than average in all of Austria. In Lower Austria, deviations in precipitation were − 36 %, in temperatures + 3.4 °C [17]. These climatic conditions most likely supported the growth of non-O1/non O-139 *V. cholerae* in these two ponds and in 7 % of the 90 EU bathing waters tested. This may also explain the high amount of *V. cholerae* (> 11,000 MPN/100 ml) present in the two swimming ponds at the time of first testing, high numbers which did not correlate with an increase in enterococci and *E. coli*, the classical indicator organisms used for bathing water surveillance.

Public health authorities have already expressed increasing concern regarding the role of climate change in driving bacterial waterborne infectious diseases [18]. Associations between environmental changes observed in the Baltic area and the recent emergence of non-O1/non O-139 *V. cholerae* infections have prompted ECDC to implement a real-time model that uses daily updated remote sensing data to map environmental suitability for *Vibrio* growth in the Baltic Sea [19]. The overall occurrence of non-O1/non O-139 *V. cholerae* infections is still low. However, the two cases of necrotizing fasciitis described here and related to bathing sites in Austria raise important questions about environmental reservoirs of non-O1/non O-139 *V. cholerae* in view of increasing extreme weather conditions. Global warming of water bodies will inevitably lead to an increased occurrence of non-O1/non O-139 *V. cholerae* and resulting problems will not be restricted to the Baltic Sea. There is a need for centralized and systematic case reporting methods. Baker-Austin et al. [18] have asked for efforts to inform at-risk groups to prevent them from recreational contact with unsafe water during periods of sustained surface water temperature warming. However, our cases reveal that presently, at-risk groups are neither properly defined, nor are the presently applied EU-parameters for testing bathing waters able to allow proper risk assessment concerning non-O1/non O-139 *V. cholerae*. Public health authorities are challenged to assess the effects of long-term climate change on pathogen growth and survival in continental fresh water bodies and to determine routes of exposure as well as the role of host susceptibility in disease emergence.
